# PAPRec: 3D Point Cloud Reconstruction Based on Prior-Guided Adaptive Probabilistic Network

**DOI:** 10.3390/s25051354

**Published:** 2025-02-22

**Authors:** Caixia Liu, Minhong Zhu, Yali Chen, Xiulan Wei, Haisheng Li

**Affiliations:** 1 Beijing Key Laboratory of Big Data Technology for Food Safety, School of Computer and Artificial Intelligence, Beijing Technology and Business University, No.33, Fucheng Road, Haidian District, Beijing 100048, China; liucaixia@btbu.edu.cn (C.L.); 2230702042@st.btbu.edu.cn (M.Z.); chenyali0601@yeah.net (Y.C.); 2 School of Logistics, Beijing Wuzi University, No.321, Fuhe Street, Tongzhou District, Beijing 101149, China; weixiulan@bwu.edu.cn

**Keywords:** 3D reconstruction, prior feature, adaptive probabilistic model, single-view image

## Abstract

Inferring a complete 3D shape from a single-view image is an ill-posed problem. The proposed methods often have problems such as insufficient feature expression, unstable training and limited constraints, resulting in a low accuracy and ambiguity reconstruction. To address these problems, we propose a prior-guided adaptive probabilistic network for single-view 3D reconstruction, called PAPRec. In the training stage, PAPRec encodes a single-view image and its corresponding 3D prior into image feature distribution and point cloud feature distribution, respectively. PAPRec then utilizes a latent normalizing flow to fit the two distributions and obtains a latent vector with rich cues. PAPRec finally introduces an adaptive probabilistic network consisting of a shape normalizing flow and a diffusion model in order to decode the latent vector as a complete 3D point cloud. Unlike the proposed methods, PAPRec fully learns the global and local features of objects by innovatively integrating 3D prior guidance and the adaptive probability network under the optimization of a loss function combining prior, flow and diffusion losses. The experimental results on the public ShapeNet dataset show that PAPRec, on average, improves CD by 2.62%, EMD by 5.99% and F1 by 4.41%, in comparison to several state-of-the-art methods.

## 1. Introduction

Three-dimensional reconstructions based on computer vision aim to generate complete 3D models of objects or scenes from images. Three-dimensional reconstruction technology has been widely used in both academia and industry due to its extensive applications in robotics [1], autonomous driving [2,3], augmented reality and virtual reality [4,5]. With the disclosure of large-scale 3D datasets [6,7,8] and the progress of deep learning, a growing number of researchers are trying to apply deep learning into the analysis and understanding of 3D objects. According to the common representation forms of 3D models, 3D reconstruction methods are mainly based on point cloud [9,10,11,12], mesh [13,14,15,16] and voxel [17,18,19,20,21,22,23,24,25,26,27]. These methods have undergone extensive study in order to provide effective tools for building 3D models.

Different from voxel and mesh representations, the point cloud representation is easy to obtain and has the characteristics of simplicity and irregularity. Previous studies [9,28,29,30] reconstruct point clouds by generative models including Generative Adversarial Networks (GANs), Variational Autoencoders (VAEs) [31], Autoregressive (AR) models, likelihood-based models and flow-based models. Although these studies have made significant progress, they have some inherent limitations. GANs often suffer from unstable training and model collapse, and AR models are not flexible. Unlike GANs, likelihood-based models and flow-based models optimize a negative log-likelihood function on training data. This function compares and measures their ability to generalize to unseen data. Moreover, likelihood-based models assign a maximum probability to all samples in the training data, so the models can theoretically cover all the patterns of the data without the problems of pattern collapse and loss of diversity that occur in GANs. Some studies utilize Neural Radiance Fields (NeRF) [32,33], which leverages the spatial and light interactions within the captured images to produce realistic 3D representations. Gaussian Splatting [34,35] further enhances this process by refining and smoothing the spatial data points, and achieves better reconstruction.

Recently, denoising diffusion probabilistic models (DDPM) [36,37] have emerged as a new class of generative models, achieving impressive performance in point cloud generation [38,39,40,41]. Based on non-equilibrium thermodynamics, DDPM is a parameterized Markov chain established by variational reasoning. It is designed to produce samples that match the real data after a finite amount of time, and the reverse process is used to learn the diffusion from the noise distribution to the real data distribution. Similarly, a point cloud can also be regarded as particles in a non-equilibrium thermodynamic system in contact with a hot bath. Therefore, the point cloud generation process can be modeled as the particle reverse diffusion process from a noise distribution to the real point cloud distribution. Subsequently, some studies combine diffusion models and Gaussian Splatting. For example, DiffGs [35] utilizes a general Gaussian generator based on latent diffusion models to reconstruct objects.

Inspired by this, we study the adaptive probabilistic network based on point cloud prior for the single-view point cloud reconstruction task. We design an adaptive decoding network consisting of a shape normalizing flow model that achieves a reversible mapping process from the Gaussian distribution to target point cloud distribution, and a diffusion model that uses the particle reverse diffusion process to transform the noise distribution into target point cloud distribution. Furthermore, we introduce an image encoder and a point encoder to obtain a shape latent vector as the noise from a single-view image before decoding. The vector is taken as the condition of a transition kernel, so as to realize high-precision 3D reconstruction.

The remainder of this paper is structured as follows. Section 2 reviews the related work on 3D reconstruction methods. Section 3 introduces the architecture and objective functions of PAPRec. Section 4 conducts comparative experiments of PAPRec with state-of-the-art methods on the ShapeNet dataset, as well as the ablation studies of PAPRec. Section 5 concludes this paper.

## 2. Related Work

### 2.1. Single-View 3D Reconstruction

In recent years, single-view 3D reconstruction has been a very challenging research hotspot in the field of computer vision. Researchers have achieved certain results by introducing prior knowledge and imposing appropriate constraints for 3D reconstruction [42,43,44,45,46,47,48,49,50]. Fan et al. [9] used a direct form of 3D model output—point cloud coordinates—which outperforms voxel-based methods. Mandikal et al. [51] trained a 3D point cloud autoencoder and then learned the mapping from the 2D image to the corresponding potential embedding space. Recently, Zhang et al. [49] proposed an encoder–decoder architecture for view-aware joint geometry and structure learning, which jointly learns multi-modal feature representations to reconstruct geometric shapes and structural details from a single-view image. Wen et al. [29] proposed a 3DAttriFlow network, which focuses on the separation and extraction of semantic attributes of different semantic levels in input images and integrates them into the process of 3D shape reconstruction. The network provides clear guidance for the reconstruction of specific attributes on 3D shapes, so as to reconstruct more accurate 3D shapes. Most of the above methods use the Chamfer distance (CD) and the Earth Mover’s distance (EMD) as loss functions to optimize neural network models. However, the loss functions have some shortcomings [52,53] because they do not guarantee that the predicted points follow the geometry of an object, and these points may exist outside the real 3D shape of an object. Zhou et al. [35] proposed a general Gaussian generator based on latent diffusion models, and achieved better reconstruction results. However, the method has a higher computational complexity and finds it difficult to preserve the details of an object.

### 2.2. Generative Models for Point Cloud

The point cloud generation task is the basis for various 3D vision tasks. Early generative models such as VAE [54], AR models [55], GAN [24,56,57,58] and flow model [29,59] have been applied to point cloud generation. However, the main drawback of these models is that they are limited to generating point clouds with a fixed number of points and lack the property of permutation invariance. Recently, researchers have proposed to treat point clouds as samples from a point distribution. Cheng et al. [55] proposed an autoregressive model that generates diverse and realistic point cloud samples under certain conditions, making good use of point-to-point correlations. Yang et al. [60] took full advantage of the reversibility of continuous normalized flow to learn two levels of distribution: the distribution of shapes and the distribution of points with a given shape. Cai et al. [52] modeled a noisy point cloud as a sample from a noisy convolution distribution, designed a network to estimate the fraction of the distribution and used the fraction to denoise the point cloud through gradient rise. Klokov et al. [61] built normalized streams with affine coupled layers to generate arbitrarily sized 3D point clouds given potential shape representations. In addition, other works developed C-Flow [62] and NeRF [32,33] to improve 3D presentation performance and obtain fine reconstruction results.

### 2.3. Diffusion Probabilistic Models

Diffusion models were applied early on in image generation and image restoration tasks [36,37,63]. Recently, these models have also shown great advantages in 3D point clouds [38,39,40,41,64,65]. The main idea is to model the reverse diffusion process of point cloud generation as a Markov chain with a specific shape. The diffusion model is easy to derive from the variable boundary of the probability of point clouds, which outperforms the previous generation model. Jiang et al. [66] proposed SDFDiff which achieves unsupervised 3D shape reconstruction. However, most of these approaches still suffer from problems, such as failing to recover occluded parts of objects completely and accurately, producing noise. Li et al. [67] used point cloud diffusion to aggregate the intermediate features of the generator into semantic labels for annotating point clouds. Lyu et al. [68] used diffusion and refinement models to complete partial point clouds. In addition, Melas et al. [69] took a single-view image and its camera parameters as inputs, and projected the local features of the image onto a partially denoised point cloud during the diffusion process, and achieved high-resolution reconstruction results. Wu et al. [70] proposed a probabilistic diffusion model that combines a hand-drawn sketch of an object and its text description to generate colored point clouds. Wei et al. [71] also applied the conditional diffusion model to the 3D reconstruction task and achieved good results. Liu et al. [72] proposed Zero-1-to-3 which uses a viewpoint-conditioned diffusion approach for 3D reconstruction from a single image. Hong et al. [73] proposed LRM which adopts a highly scalable transformer-based architecture to predict a neural radiance field from an input image.

## 3. PAPRec

**Figure 1:**
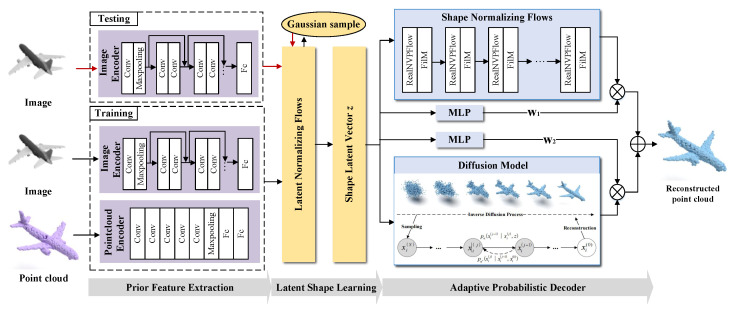
The architecture of our proposed PAPRec. In the training stage, PAPRec encodes a single-view image and its corresponding 3D shape into feature distributions. PAPRec then utilizes a latent normalizing flow to fit the distributions and obtain a shape latent vector. PAPRec finally introduces an adaptive probabilistic network, which consists of a flow model and a diffusion model for fully learning the global and local features of objects, to decode the latent vector as a complete 3D point cloud. In the testing stage, PAPRec utilizes the trained image encoder, latent normalizing flow and adaptive decoder to reconstruct the 3D point cloud from a single-view image.

As shown in Figure 1, PAPRec contains three parts: prior feature extraction, latent shape learning and adaptive probabilistic decoding. PAPRec incorporates 3D prior knowledge and adopts an adaptive probabilistic decoder to learn the global and local information of an object, which is conductive to capture the mapping between an input image and the corresponding 3D point cloud. The training and testing details are shown in Algorithm 1.
**Algorithm 1:** Training and testing process
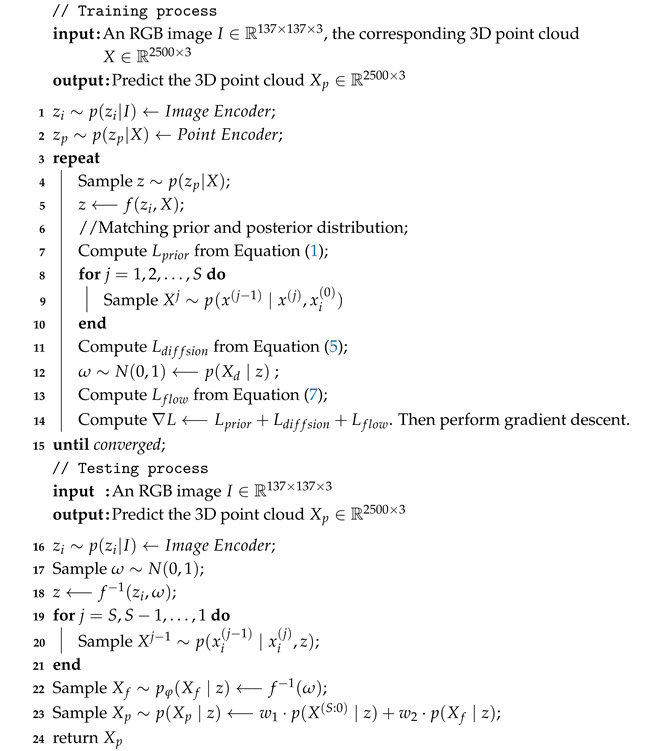


### 3.1. Prior Feature Extraction

In the single-view 3D point cloud reconstruction task, we regard a point cloud as a conditional probabilistic distribution. As a result, the goal is to learn a distribution p(X|I), where *I* is an input image. In order to obtain rich cues given few inputs, we introduce a point cloud-based prior to guide the learning of the mapping between the input and the real point cloud during training.

Specifically, we utilize a point cloud encoder to parameterize a real point cloud *X* as a feature vector $z_{p}$. The encoder consists of five convolutional layers with feature channels of {3, 64, 128, 256, 512}, a max-pooling layer and two fully connected layers with 512 channels. Meanwhile, we also utilize an image encoder consisting of ResNet18 [74] to parameterize an input image *I* as a feature vector $z_{I}$. Then, we obtain the point cloud and image latent conditional distributions $p(z_{p}|X)$ and $p_{\theta }\left(z_{i}|I\right)$. $p(z_{p}|X)$ and $p(z_{i}|I)$ are modeled as normal distributions.

### 3.2. Latent Shape Learning

In order to fully learn the latent distribution of an object, we introduce a latent normalizing flow model *f* to approximate $p(z_{p}|X)$ and $p(z_{i}|I)$. The flow model provides a trainable bijector that maps isotropic Gaussian distribution to a complex distribution.

The latent normalizing flow model consists of 14 affine coupling layers, each of which is followed by batch normalization and the ReLU activation function. The channels are set as {128, 256, 256, 128}. The model finally outputs a shape representation $z\in R^{512}$ for 3D generation tasks and single-view 3D reconstruction tasks.

The prior loss $L_{prior}$ is used to minimize the distance between the image distribution and the point cloud distribution, and it is defined as follows:

(1) $$\begin{array}{cc} L_{prior} & =-H\left(p\right)-E_{z∼p(z_{p}|X)}\left[\log(p\left(z_{i}|I\right))\right]\\ \; & =-H\left(p\right)-E_{z∼p(z_{p}|X)}\left[\log(N\left(f^{-1}\left(z_{i}\right)\right)\right)+\log(\det|J_{f^{-1}}(z_{i})|)], \end{array}$$

where *X* denotes the true point cloud. H(·) denotes the calculation of the approximate posterior entropy loss, and it can prevent the latent distribution from being either too concentrated or too dispersed, and also prevent the model from overfitting the training data. E(·) denotes the calculation of the mathematical expectation. N(·) denotes the sampling from a Gaussian distribution, $f^{-1}\left(z_{i}\right)$ denotes a trainable bijector with a normalized flow conditioned on image eigenvectors, det(·) denotes the calculation of the determinant, and J(·) denotes the calculation of Jacobian determinant.

In the training process, we combine the prior distribution of the image with the posterior distribution of the point cloud, and fit the posterior and the prior as Gaussian distributions, thus improving the generation performance. Therefore, we obtain the distribution of the shape latent vector p(z):

(2) $$p\left(z\right)=p_{\omega }\left(\omega \right)\cdot \det{\left|\frac{\partial f}{\partial \omega }\right|}^{-1},$$

where $p_{\omega }\left(\omega \right)$ denotes the isotropic Gaussian distribution N(0,1).

### 3.3. Adaptive Probabilistic Decoder

We design a decoder which is an adaptive probabilistic model consisting of a diffusion model and a shape normalizing flow for learning the local and global information of objects. In particular, we set adaptive weights $w_{1}$ and $w_{2}$ for the two models to represent the entire shape of an object. The weights are obtained by two Multi-Layer Perceptrons (MLPs), each of which contains two fully connected layers with 512 channels, batch normalization, and the Swish activation function. Therefore, we can express the conditional distribution of the predicted point cloud as $p(X_{p}|z)$:

(3) $$p\left(X_{p}|z\right)=w_{1}\cdot p\left(X^{\left(S:0\right)}|z\right)+w_{2}\cdot p\left(X_{f}|z\right),$$

where, the former and the latter represent the diffusion model and the shape normalizing flow model, respectively. *S* is the diffusion step.

For the diffusion model, it is a variant of MLP consisting of a series of concatenation and squash layers to achieve the reverse diffusion kernel. The point cloud is represented as $X^{\left(j\right)}={\left\{x_{i}^{\left(j\right)}\right\}}_{i=1}^{N}$, where $x_{i}^{\left(j\right)}$ represents the *i*th point $x_{i}$ at the *j*th step, which is regarded as a set of particles in the evolutionary thermodynamic system. The initial point $x_{i}^{\left(0\right)}$ can be regarded as a sample independent of the true point distribution. Over time, the points gradually spread out into a chaotic set of points, which converts the original meaningful distribution of points into a noise distribution. The reverse process aims to recover the desired shape from the input noise. The process can be formulated as follows:

(4) $$\begin{array}{cc} p(X^{\left(S:0\right)}|z) & =\prod_{i=1}^{N}p\left(x_{i}^{\left(S:0\right)}|z\right)\\ \; & =\prod_{i=1}^{N}\left[p\left(x_{i}^{\left(S\right)}\right)\prod_{j=1}^{S}p\left(x_{i}^{\left(j-1\right)}|x_{i}^{\left(j\right)},z\right)\right]\\ \; & =\prod_{i=1}^{N}\left[p\left(x_{i}^{\left(S\right)}\right)N\left(x_{i}^{\left(j-1\right)}|\mu \left(x_{i}^{j},j,z\right),\beta_{j},1\right)\right], \end{array}$$

where the starting distribution $p(x_{i}^{\left(S\right)})$ in the reverse diffusion process is set to the standard normal distribution N(0,1), and μ is the estimated value by the neural network. $\beta_{1},\ldots ,\beta_{j},\ldots ,\beta_{S}$ are the variance scheduling parameters controlling the rate of the diffusion process, and they are set by the common linear scheduling formula $\beta_{j}=\beta_{1}+\left(\beta_{S}-\beta_{1}\right)\times \left(j-1\right)/\left(S-1\right)$.

The goal of training the reverse diffusion process is to maximize the log-likelihood of the point cloud. However, the direct optimization of the log-likelihood is difficult to solve. Therefore, we maximize its variational lower bound, and the diffusion loss $L_{diffusion}$ is defined as follows:

(5) $$\begin{array}{c} \begin{array}{cc} L_{diffusion}= & E[\sum_{j=2}^{S}\sum_{i=1}^{N}D_{\mathrm{KL}}\left(p\left(x_{i}^{\left(j\right)}|x_{i}^{\left(j-1\right)},x_{i}^{\left(0\right)}\right)|\;|p\left(x_{i}^{\left(j-1\right)}|x_{i}^{\left(j\right)},z\right)\right)-\\ \; & \;\sum_{i=1}^{N}\log p\left(x_{i}^{\left(0\right)}|x_{i}^{\left(1\right)},z\right)D_{\mathrm{KL}}\left(p\left(z|X^{\left(0\right)}\right)|\;|p_{\omega }\left(\omega \right)\cdot {\left|\left(\det\frac{\partial f}{\partial \omega }\right)\right|}^{-1}\right)], \end{array} \end{array}$$

where $D_{\mathrm{KL}}\left(\cdot \right)$ denotes the calculation of KL divergence and and this divergence is used to measure the difference between two probability distributions, which helps the reverse diffusion process to better restore the real point cloud state.

For the shape normalizing flow model, we use the same network as the latent normalizing flow, and the network consists of 14 RealNVPFlow and FilM layers [75]. The reconstructed 3D point cloud distribution of the model $p(X_{f}|z)$ is defined as follows:

(6) $$\begin{array}{c} p\left(X_{f}|z\right)=N\left(f_{\varphi }^{-1}\left(X_{f}\right),\mu \left(z\right),\Sigma \left(z\right)\right)\left|\det(\frac{\partial f}{\partial X_{f}^{T}})\right| \end{array}$$

The loss of the shape normalizing flow $L_{flow}$ is defined as follows:

(7) $$\begin{array}{cc} L_{flow} & =E_{X_{f}∼p\left(X_{f}|z\right)}\left[-\log(p\left(X_{f}|z\right))\right]\\ \; & =-E_{X_{f}∼p\left(X_{f}|z\right)}\left[\log(N\left(f^{-1}\left(z\right)\right))\right]-\log\left(\det\left|J_{f^{-1}}\left(X_{f}\right)\right|\right), \end{array}$$

where $X_{f}^{T}$ denotes the transpose of $X_{f}$.

Our PAPRec is optimized in an end-to-end way by minimizing the total loss *L*, which is defined as follows:

(8) $$L=L_{prior}+L_{diffusion}+L_{flow}$$

## 4. Experiments

In this section, we describe the datasets, evaluation metrics and the experimental implementation details. We then discuss and analyze the comparison with the most relevant works based on quantitative and qualitative results.

### 4.1. Dataset

Following [61], we use the ShapeNet dataset [76] to evaluate the performance of PAPRec. In particular, for the 3D point cloud generation task, we adopt 51,127 shapes from 55 categories in the ShapeNet dataset. For the single-view 3D reconstruction task, we adopt 13 object categories {airplane, bench, cabinet, car, chair, monitor, lamp, speaker, rifle, sofa, table, telephone, vessel} from ShapeNet provided by 3D-R2N2 [77], and obtain the data pairs of images and point clouds. The above datasets are randomly divided into 80% training set and 20% testing set.

### 4.2. Evaluation Metric

In order to compare the reconstruction performance of different methods on public datasets, we use the Chamfer distance (CD) and the Earth Mover’s distance (EMD) to measure the reconstruction quality. In addition, we also use the F1-score to evaluate the reconstruction accuracy at the point cloud level.

CD is used to measure the distance between two point clouds by calculating the square distance between each point *p* in the predicted point cloud $X_{p}$ set and the nearest neighbor of each point *q* in the true point cloud *X* set:

(9) $$\mathrm{CD}=\min_{p\in X_{p}}{∥p-q∥}^{2}+\min_{q\in X}{∥p-q∥}^{2}$$

The lower (denoted by ↓) the CD is, the better the reconstructed 3D shape is.

EMD is used to measure the heterogeneity between two multi-dimensional distributions in a certain feature space by calculating the point-to-point L1 distance between two point clouds.

(10) $$\mathrm{EMD}=\min_{\phi :X_{p}⟶X}\sum_{p\in X_{p}}∥p-\phi (p)∥$$

The lower (denoted by ↓) the EMD is, the better the reconstructed 3D shape is.

The F1-score is used to measure the correct percentage of reconstructed points by calculating the harmonic average of accuracy *P* and recall *R* at a threshold.

(11) $$F1=\frac{2\times P\times R}{\left(P+R\right)}$$

The higher (denoted by ↑) the F1 is, the better the reconstructed 3D shape is.

### 4.3. Parameter Setting

We optimize all the losses under the Pytorch (https://pytorch.org/) framework using the Adam algorithm [78] with default values. The learning rate is set to $2.56\times 10^{-4}$ by following [33]. The size of a point cloud is set as N=2048 (for the 3D generation task) and N=2500 (for the single-view reconstruction task). The diffusion step is set as S=200 to balance between computational resources and the generation effect. The variance scheduling parameters are set as $\beta_{1}=0.0001,\;\beta_{S}=0.05$. The former is to preserve the features and structure of the original data well and the latter is so that the data reach a certain level of noise, but do not transform completely into random noise without any pattern.

### 4.4. Comparisons with State-of-the-Art Methods

**Figure 2:**
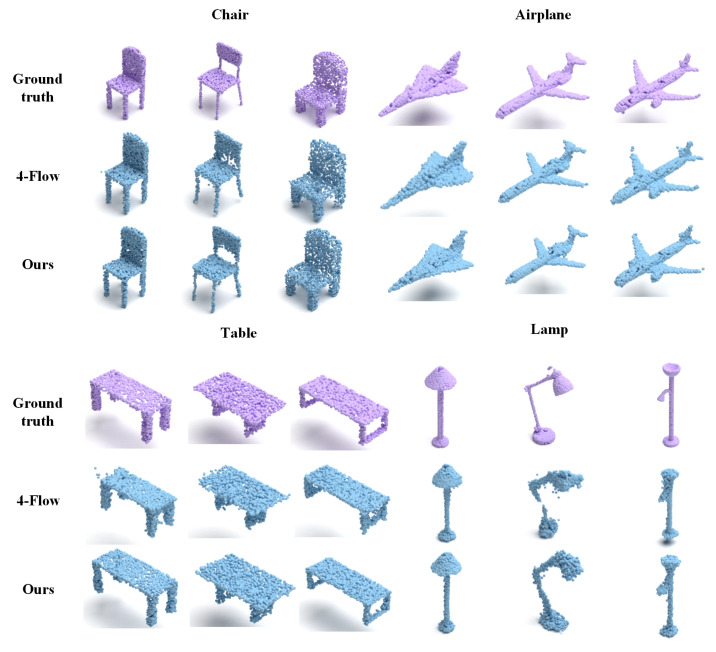
The qualitative comparison results of different autoencoders. Our reconstructed chairs and airplanes exhibit higher accuracy, while the reconstructed tables and lamps are more complete. This clearly demonstrates that PAPRec has greater effectiveness and a stronger capacity to represent diverse shapes.

**Figure 3:**
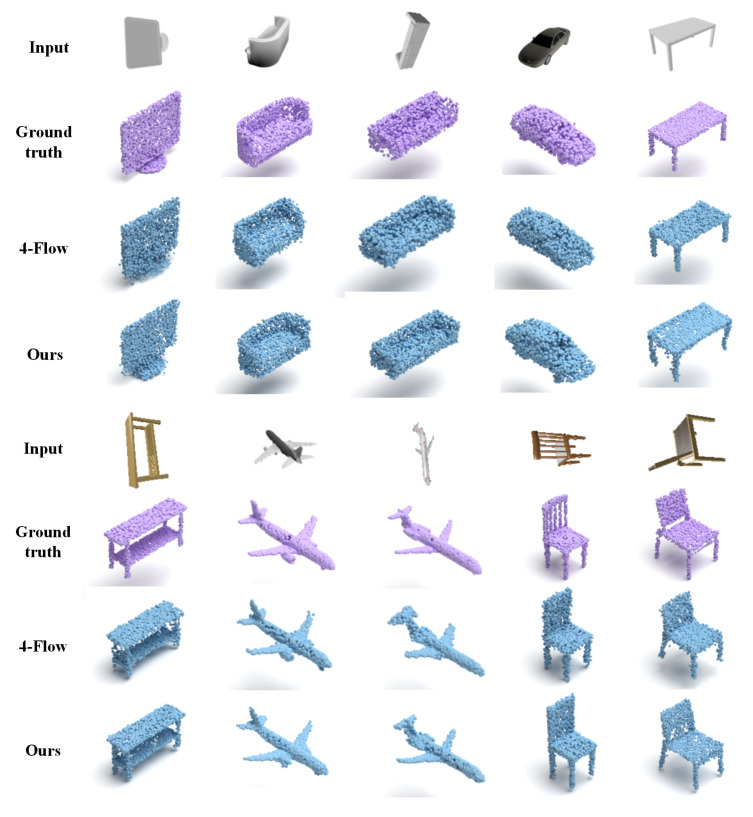
The qualitative comparison results of the single-view reconstruction methods. Our reconstructed monitors, benches and cars exhibit more details, while the reconstructed tables, planes and chairs are more accurate. This clearly demonstrates that PAPRec has better robustness.

We conduct two kinds of experiments to evaluate the performance of PAPRec: a 3D generation task and a single-view 3D reconstruction task. The former aims to demonstrate the advantage of our autoencoder, and the comparison results are shown in Figure 2 and Table 1. The latter is designed to demonstrate our performance in the single-view reconstruction task, and the comparison results are shown in Figure 3 and Table 2 and Table 3.

Specifically, for the 3D generation task, we compare the current mainstream generative models, such as the GAN [58] and flow-based [33,60,61] methods. From Table 1, it is observed that our PAPRec outperforms the others in both CD and EMD. From Figure 2, the improvement of the lamp category is particularly obvious, which indicates that PAPRec has greater effectiveness and a stronger capacity to represent diverse shapes.

For the single-view reconstruction task, we compare several reconstruction methods [33,42,43,61,79]. Among them, DPF [61] is closest to us, and it also regards the point cloud as the distribution of samples and uses the normalized flow model for decoding. 4-Flow [33] realizes the parallel decoding of four normalized flows to reconstruct the overall shape and makes a slight improvement on DPF, showing better optimization. However, 4-Flow cannot generate good details. From Figure 3 and Table 2 and Table 3, it can be ssen that PAPRec is superior to the current mainstream single-view reconstruction methods in both quantitative and qualitative results. PAPRec owes its superiority to two key aspects. It uses a diffusion probability decoding network for the gradual refinement of point cloud reconstruction, and integrates a flow model to learn features of the point cloud’s overall distribution. Moreover, PAPRec uses 3D point cloud priors to guide single-view image reconstruction and accurately capture local and global information during 3D point cloud generation.

### 4.5. Ablation Study

We conduct the experiments to analyse the importance of each component from PAPRec. We denote

$$\begin{array}{cc} \;\;\text{-Prior:} & \text{PAPRec without the point cloud learning during the training.}\\ \;\;\text{-Flow:} & \text{PAPRec without the shape normalizing flow.}\\ \;\;\text{-Diffusion:} & \text{PAPRec without the diffusion model.}\\ \;\;\text{-Fixed decoder:} & \text{PAPRec with fixed weights in the decoder.} \end{array}$$

**Figure 4:**
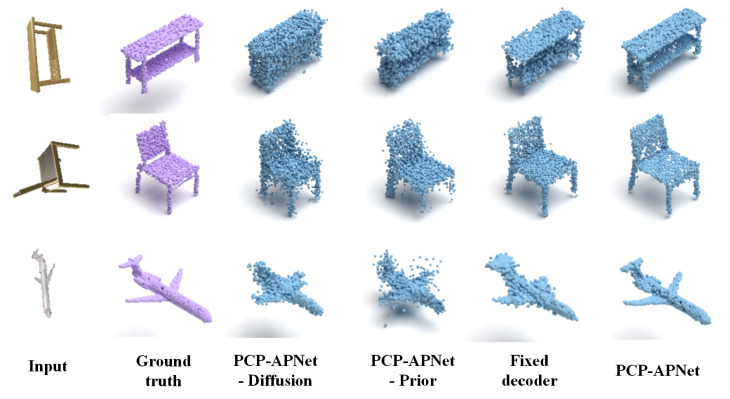
The qualitative comparison results of ablation study. ‘-Prior’, ‘-Flow’ and ‘-Diffusion’ denote PAPRec without the point cloud learning, the shape normalizing flow and the diffusion model, respectively. ‘Fixed decoder’ denotes PAPRec adopts fixed weights in the decoder. The full model achieves superior performance in handling noise and reproducing details.

Table 4 and Figure 4, respectively, present the quantitative and qualitative results of the ablation study. As can be seen from Figure 4, when only image features are employed as the shape latent vector for decoding, the reconstruction effect is evidently subpar. For instance, when dealing with a chair that has severe self-occlusion, the model fails to reconstruct the details of its legs. Similarly, for other object categories, the reconstruction results have noise. Moreover, neither a single flow model nor a diffusion model alone can achieve faithful reconstruction; instead, they tend to generate noise. Additionally, while a non-adaptive probabilistic decoder can reconstruct the general shape, it performs poorly in capturing details. Thus, both the quantitative and qualitative results verify that the full model is innovative and effective.

## 5. Conclusions

We propose a novel framework, PAPRec, that reconstructs the complete 3D point cloud of an object from a single-view image. Different from the proposed methods, the main contributions of our work can be summarized as follows:

- PAPRec introduces point cloud models to guide a corresponding single-view image to express the potential space of an object by optimizing a prior loss. This makes up for insufficient feature expression of a single image and effectively improves the ambiguity reconstruction.
- PAPRec intorduces an adaptive probabilistic decoding network. The network adopts adaptive weights to combine two models and utilizes a flow loss and a diffusion loss to optimize them. One model is a shape normalizing flow model, which is used to interpret the overall distribution of a point cloud for obtaining global information. Another model is diffusion model, which is used to extract local information. The network is inductive to stable training and adaptive constrains, thus generating high-quality reconstruction.
- The experimental results show that PAPRec improves average CD by 2.62%, EMD by 5.99% and F1 by 4.41% on the ShapeNet dataset for the single-view 3D reconstruction.

Although PAPRec achieves better reconstruction by innovatively integrating 3D prior guidance and the adaptive probability network, there is still room for improvement in generating details. Based on the fact that accurate 3D models are very important for some high-level multimedia applications, we will conduct more research on improving the reconstruction accuracy by using prior-based diffusion models.

## Figures and Tables

**Table 1:** The quantitative comparison results of different autoencoders on the full ShapeNet dataset. The bold numbers denote the best results.

Method	CD ($1\times 10^{-3}$) (↓)	EMD ($1\times 10^{-2}$) (↓)	F1 (τ = $1\times 10^{-4}$) (↑)
4-Flow [33]	6.88	4.80	34.75
DPF [61]	7.07	7.70	34.50
1-GAN-CD [58]	9.18	7.70	-
1-GAN-EMD [58]	9.18	5.30	-
PointFlow [60]	7.54	5.18	32.30
AtlasNet [43]	5.66	5.81	-
PAPRec	**5.44**	**3.79**	**37.29**

**Table 2:** The quantitative comparison results of a single-view 3D reconstruction on the 13 object categories of ShapeNet. The bold numbers denote the best results.

Method	CD ($1\times 10^{-3}$) (↓)	EMD ($1\times 10^{-2}$) (↓)	F1 (τ = $1\times 10^{-4}$) (↑)
4-Flow [33]	5.66	11.18	52.30
DPF [61]	5.80	11.17	52.12
Pixel2Mesh [42]	5.91	13.80	-
DCG [79]	6.35	18.94	-
AtlasNet [43]	5.34	12.54	-
PAPRec	**5.20**	**10.50**	**54.42**

**Table 3:** The quantitative comparison results of single-view reconstruction on each category of ShapeNet. The bold numbers denote the best results.

Category	CD ($1\times 10^{-3}$) (↓)		EMD ($1\times 10^{-2}$) (↓)		F1 (τ = $1\times 10^{-4}$) (↑)	
	Ours	4-Flow	Ours	4-Flow	Ours	4-Flow
airplane	**1.72**	2.79	**7.97**	8.98	**80.31**	78.54
bench	**3.57**	4.52	**9.96**	10.12	**66.91**	63.74
cabinet	**4.68**	5.83	**10.72**	11.29	**40.62**	37.63
car	**3.16**	3.98	**10.01**	10.52	**49.01**	44.54
chair	**4.80**	5.45	**10.99**	11.40	**51.65**	46.19
monitor	**8.93**	9.15	**11.41**	12.42	**52.31**	45.55
lamp	**14.54**	16.53	**14.89**	16.90	**54.28**	43.60
speaker	**10.35**	12.98	**13.59**	14.69	**31.23**	26.62
rifle	**1.32**	2.28	**7.27**	8.62	**86.80**	83.66
sofa	**5.32**	6.25	**10.43**	11.62	**45.28**	38.14
table	**4.46**	5.95	**10.39**	11.53	**58.80**	52.57
telephone	**2.20**	3.73	**9.71**	10.51	**60.30**	58.57
vessel	**2.31**	4.95	**9.04**	10.32	**69.42**	61.73
mean	**5.18**	6.49	**10.49**	11.46	**57.45**	52.39

**Table 4:** Quantitative results of ablation study. ‘-Prior’, ‘-Flow’ and ‘-Diffusion’ denote PAPRec without the point cloud learning, the shape normalizing flow and the diffusion model, respectively. ‘Fixed decoder’ denotes PAPRec adopts fixed weights in the decoder. The bold numbers denote the best results.

Method	CD ($1\times 10^{-3}$) (↓)	EMD ($1\times 10^{-2}$) (↓)	F1 (τ = $1\times 10^{-4}$) (↑)
-Prior	10.07	15.02	41.08
-Flow	8.40	15.70	50.99
-Diffusion	9.87	13.73	41.26
Fixed decoder ($w_{1}=w_{2}=\frac{1}{2}$)	8.47	16.25	42.24
PAPRec (Adaptive decoder)	**5.20**	**10.50**	**54.42**

## Data Availability

The details of the data are shown in Section 3.1.
